# Associations of specific types of fruit and vegetables with perceived stress in adults: the AusDiab study

**DOI:** 10.1007/s00394-022-02848-5

**Published:** 2022-03-20

**Authors:** Simone Radavelli-Bagatini, Marc Sim, Lauren C. Blekkenhorst, Nicola P. Bondonno, Catherine P. Bondonno, Richard Woodman, Joanne M. Dickson, Dianna J. Magliano, Jonathan E. Shaw, Robin M. Daly, Jonathan M. Hodgson, Joshua R. Lewis

**Affiliations:** 1 Institute for Nutrition Research, School of Medical and Health Sciences, Edith Cowan University, Perth, WA Australia; 2 Medical School, The University of Western Australia, Perth, WA Australia; 3 Flinders Centre for Epidemiology and Biostatistics, Flinders University, Adelaide, SA Australia; 4 School of Arts and Humanities (Psychology), Edith Cowan University, Perth, WA Australia; 5 Diabetes and Population Health, Baker Heart and Diabetes Institute, Melbourne, VIC Australia; 6 School of Public Health and Preventive Medicine, Monash University, Melbourne, VIC Australia; 7 Clinical Diabetes and Epidemiology, Baker Heart and Diabetes Institute, Melbourne, VIC Australia; 8 Institute for Physical Activity and Nutrition, School of Exercise and Nutrition Science, Deakin University, Geelong, VIC Australia; 9 Centre for Kidney Research, Children’s Hospital at Westmead, School of Public Health, Sydney Medical School, The University of Sydney, Sydney, NSW Australia

**Keywords:** AusDiab, Australian adults, Perceived stress, Types of fruit and vegetable intake

## Abstract

**Purpose:**

Higher total fruit and vegetable (FV) intakes have been associated with lower perceived stress. The relationship between specific types of FV and perceived stress remains uncertain. The aims of this cross-sectional study were to explore the relationship between consumption of specific types of FV with perceived stress in a population-based cohort of men and women aged ≥ 25 years from the Australian Diabetes, Obesity and Lifestyle (AusDiab) Study.

**Methods:**

Dietary intake was assessed using a validated Food Frequency Questionnaire (*n* = 8,640). Perceived stress was evaluated using a validated Perceived Stress Questionnaire, with values ranging 0–1 (lowest to highest). High perceived stress cut-offs of ≥0.34 for men and ≥0.39 for women were obtained from the highest quartile of the perceived stress score for each sex. Multivariable-adjusted logistic regression was performed to investigate the associations.

**Results:**

The mean age of participants (50.1% females) was 47.8 (SD 15) years. Persons in the highest, versus lowest, quartiles of apples and pears, orange and other citrus, and banana intakes had a significantly lower odds (24–31%) of having high perceived stress. Similarly, persons with higher intakes of cruciferous, yellow/orange/red, and legume vegetables had significantly lower odds (25–27%) of having high perceived stress.

**Conclusion:**

In Australian adults, a higher consumption of apples and pears, oranges and other citrus, and bananas, as well as cruciferous, yellow/orange/red, and legume vegetables were associated with lower odds of having high perceived stress. The recommendations of “eating a rainbow” of colours may assist in preventing and/or reducing perceived stress.

**Supplementary Information:**

The online version contains supplementary material available at 10.1007/s00394-022-02848-5.

## Introduction

Perceived stress can be defined as the thoughts and feelings an individual has about how much stress they experience in response to stressful life events [1]. For this manuscript, the term “stress” will be used to represent “perceived stress” for simplicity. Whereas some stress is part of humans life, long-term elevated stress can adversely affect health and lead to mental [2] and physical disorders [3], such as depression [4] and cardiovascular disease (CVD) [5, 6]. Mental health problems affect 1 in 5 people worldwide [7]. In the United States, mental health problems are estimated to cost the global economy over US$1 trillion each year [8]. Therefore, there is a need for public health strategies to prevent and/or reduce stress levels.

In addition to the benefits of an active lifestyle, growing evidence indicates that diet [9, 10], in particular high consumption of fruit and vegetables (FV), can positively impact mental health [11]. Findings from a systematic review [12] that included 61 observational studies showed a positive relationship between FV intake and several mental health-related outcomes, such as depression, anxiety, stress, mood, self-esteem, creativity, and quality of life. However, to date, only a few studies have investigated the association of specific types of FV [12] with mental health [13–17], with only one study considering stress [13]. Noteworthy, due to the varying nutrient composition of different types of FV, it is possible that specific fruits and/or vegetables may provide additional benefits. To date, however, this remains unknown. For example, specific fruit and/or vegetable types may be richer sources of some vitamins, minerals, carotenoids and flavonoids [18], which may potentially alleviate stress levels [19].

Further studies investigating whether specific FV types may offer additional benefits for perceived stress are warranted. It is noteworthy that studies in this area have typically focussed on depression and other mental health issues [12], and had a relatively small number of participants within specific demographics (e.g. middle-aged persons, students) [13–17]. Therefore, a broader and more representative sample of the adult lifespan should be explored. Previously, we demonstrated an association between higher total FV consumption and lower perceived stress in the Australian Diabetes, Obesity and Lifestyle Study (AusDiab) cohort [20]. However, it remains unclear if specific types of fruits and/or vegetables may provide greater benefits when considering perceived stress. Therefore, the aims of this study were to further investigate the cross-sectional associations of FV types with perceived stress, in this cohort of Australian adults.

## Materials and methods

### Study population

Participants included in this study were part of the AusDiab study, a national population-based survey of men and women aged ≥ 25 years, recruited in 1999–2000 from 42 randomly selected census collector districts across Australia. Detailed methods have been published elsewhere [21]. A total of 20,347 Australian adults completed a household interview at baseline (1999–2000), and 11,247 of those attended a clinical examination (5,049 men; 6,198 women) in the original cohort study. For the present cross-sectional study, those with missing data for exposures, outcome and confounding variables were excluded. This comprised people who did not complete the baseline perceived stress questionnaire (*n* = 1,697) and the food frequency questionnaire (FFQ, *n* = 148), as well as individuals with at least one missing value for confounding factors (*n* = 436), those with implausible energy intake (<3300 or >17,500 kJ in men and <2,500 kJ or >14,500 kJ in women) (*n* = 275) [22], and pregnant women (*n* = 51). In total, 8,640 participants were included in the present study, after excluding missing data (*n* = 2,607). A flow diagram is shown in Online Resource 1.

### Perceived stress assessment

A validated 30-item Perceived Stress Questionnaire (PSQ) [23] was used to assess perceived stress levels. Participants reported their feelings related to stressful situations (i.e., “you feel rested”, “you feel that too many demands are made on you”, “you are irritable or grouchy”) over the previous 12 months. Response options ranged from 1 to 4 on a four-point Likert scale. All positive questions (Questions 1, 7, 10, 13, 17, 21, 25, 29), such as “you feel rested”, were reverse scored (i.e., 1 [“usually”] to 4 [“almost never”]) and all other [negative] questions were scored from 1 (“almost never”) to 4 (“usually”). A perceived stress index was calculated from the raw scores which ranged from a minimum of 30 to a maximum of 120, using the formula: perceived stress index = (raw score – 30)/90 [23], with a higher index indicating greater perceived stress levels. As men are known to have lower perceived stress than women [24], individuals with high perceived stress were identified based on a specific cut-point derived from participants in the highest quartile of the perceived stress for each sex (e.g. high perceived stress; ≥0.34 and ≥0.39 for men and for women, respectively).

### Dietary intake

Dietary intake was assessed using a validated 74 food item FFQ developed by the Cancer Council of Victoria [25], with participants recalling their usual intakes over the previous 12 months. Nutrient intakes were estimated based on frequency of consumption and an estimation of usual serving size. Energy intake (kcal/day) was calculated based on individuals total reported food consumption [25]. Alcohol consumption was assessed using the questions about consumption of alcoholic beverages [25], with frequency of alcohol intake ranging from ‘never’ to ‘every day’.

#### Fruit and vegetable types

Fruit types were grouped into three different categories [26]: apples and pears; oranges and other citrus fruits; and bananas. These are the most common fruit consumed in Australia [27]. Fruit juice, tinned and dried fruit were not included as these types of fruit should be consumed only occasionally as substitutes for fresh fruit, according to The Australian Dietary Guidelines [28]. The reason for this is the high sugar content of dried fruit and some fruit juices, as well as the absence of dietary fibre in juices [28]. Types of vegetables were classified into five groups according to their phytochemical properties [29–34] and based on the Australian Dietary Guidelines [28]: cruciferous vegetables (cabbage, Brussels sprouts, cauliflower, and broccoli); allium vegetables (onion, leek, and garlic); yellow/orange/red vegetables (tomato, capsicum, carrot, beetroot and pumpkin); leafy green vegetables (lettuce and other salad greens, celery, silver beet, and spinach); and legumes (peas, greens beans, bean sprouts and alfalfa sprouts, baked beans, soy beans, soy bean curd and tofu, and other beans). “Potato, roasted and fried, including hot chips” was excluded as these are not considered part of a healthy diet. This method of classifying vegetable types has been used extensively [35–38]. Intake for each FV type was calculated (g/day) and subsequently categorised into quartiles. Vegetable diversity (number of different vegetables consumed daily) was obtained from the question “How many different vegetables do you usually eat in a day?”, as described in previous studies [39–41].

### Baseline demographic and clinical assessment

Demographic information was collected at a household interview and included age (date of birth), sex (male/female), relationship status (de facto, married, separated, divorced, widowed, never married), education level (never to some high school, completed University or equivalent) and average weekly income (according to six categories: $0–199, $200–399, $400–599, $600–799, $800–1499, and >$1500). Area-based socio-economic status was assessed based on the 5-yearly census [42] from 1999, using the socio-economic index for areas (SEIFA) which takes into consideration the social and economic conditions per geographic area.

Following the household interview, anthropometric assessments were performed at the clinic visits [21, 43] including: height, measured using a stadiometer to the nearest 0.5 cm without shoes; and weight, measured using a mechanical beam balance without shoes and excess clothing to the nearest 0.1 kg [44]. Body mass index (BMI) was obtained by dividing weight (kg) by height in squared metres.

The Active Australia Survey Questionnaire was used to assess physical activity in the previous 7 days (total minutes per week) [45]. Total physical activity (min/week) was obtained by summing the total walking time (if continuous for ≥10 min) and/or performing moderate-intensity exercise, plus the time performing vigorous-intensity exercise (this was multiplied by two) and then added to the time [41].

An interviewer-administered general questionnaire was used to assess smoking status [21], with participants being classified as current smokers (smoking at least daily), ex-smokers (smoked less than daily for at least the last 3 months), or never smokers (smoked < 100 cigarettes during life) [46].

Glucose tolerance status was based on self-report of diagnosed diabetes mellitus (DM) and on plasma glucose levels, and was categorised as known DM, undiagnosed DM, impaired fasting glucose, impaired glucose tolerance, normal glucose levels. Prevalent CVD was assessed using self-reported history of CVD (yes/no) [47]. These variables were included as confounding factors in the model due to the likelihood of individuals with diabetes and a history of CVD to have been counselled on diet and lifestyle, and therefore may have changed their dietary habits.

To further explore whether diet quality could be a confounding factor in the present study, a Dietary Guideline Index (DGI) [48] was included in the analyses. The DGI includes 15 components based on the Australian Dietary Guidelines [49, 50] as previously reported [48]. Each component was given a score ranging from 0 to 10 points (from lowest to optimal intakes), with total scores ranging from 0 to 150. Higher scores indicate greater adherence to the dietary guidelines and better diet quality [48].

### Statistical analysis

Statistical significance was set at *p* ≤ 0.05 (two-tailed) for all tests. Statistical analyses were performed in Stata (Stata Statistical Software: Release 15. College Station, TX: StataCorp LLC) [51] using the suite of survey commands. The main exposures of this study were intakes of the eight types of FV (continuous [per SD] and categorical [quartiles]) and the primary outcome was the perceived stress index. Logistic regression commands for complex survey designs were performed to investigate the association between the exposures and high perceived stress (highest quartiles in men and women). Linear regression commands for complex survey designs were used to test for differences in perceived stress (continuous) with the exposures of interest. The complex survey commands adjusts standard errors and incorporates stratification weights thereby providing population-level estimates [21]. Two models of adjustment were used in the statistical analysis for each of the above analyses: (1) unadjusted; and (2) adjusted for age, sex, BMI, energy intake, relationship status, physical activity levels, level of education, SEIFA, smoking status, self-reported history of CVD, and diagnosis of diabetes based on plasma glucose levels. Participants with missing data for exposure, outcome and confounding variables were excluded from the analyses.

#### Sensitivity and interaction analyses

In the sensitivity analyses, DGI was added to the multivariable-adjusted model to further explore whether a healthy diet could be a confounding factor. When a specific fruit type was associated with lower odds of high perceived stress, we also considered a model where we adjusted for ‘all other fruit’ in the multivariable-adjusted logistic regression model. A similar analysis was performed for vegetable types. Interaction terms for FV types vs. age and FV types vs. sex were assessed to investigate potential effect modification for perceived stress in multivariable-adjusted models, with both FV types and perceived stress index entered as continuous variables.

#### Additional analysis

The Spearman rank correlation test (*r*_s_) for non-parametric variables was used to assess the correlation between vegetable diversity (number of different vegetables consumed per day) and intake (total amount in g/day). We sought to assess if vegetable diversity was associated with high perceived stress, independent of total vegetable intake. Variable inflation factors (VIF) were examined in the aforementioned multivariable-adjusted model to assess for collinearity.

## Results

### Baseline characteristics

Table 1 shows the baseline characteristics of the 8,640 participants in total and by stress levels (lower vs. high stress). A total of 50.1% (*n* = 4,747) were women and the mean (±SD) age of participants was 47.8 ± 15 years. Approximately, 16.3% were current smokers and 6.7% had a history of CVD. Normal glucose levels were present in 77.1% of the participants. Average perceived stress index was 0.28 ± 0.16 (mean ± SD). Perceived stress was 0.20 ± 0.10 in the three lowest stress quartiles, and 0.49 ± 0.10 in the high stress group. Participants with higher stress levels tended to be younger, less physically active with a higher energy intake and higher levels of education (all *p* < 0.05) compared to the lower stress groups. Relationship status, smoking habits, history of CVD and diabetes were also significantly different between the groups with high and lower stress levels (all *p* < 0.01).

**Table 1:** Clinical and demographic characteristics of all participants and by perceived stress

	All	Lower stress	High stress
	*n* = 8640	*n* = 6456	*n* = 2184
Age (years)	47.8 ± 15.0	49.7 ± 16.0	43.0 ± 11.1
Sex (women), *n* (%)	4747 (50.1)	3601 (51.7)	1146 (46.3)
BMI (kg/m^2^)	26.6 ± 4.8	26.6 ± 4.9	26.6 ± 4.5
Energy intake (kcal/d)	2042 ± 697	2002 ± 704	2143 ± 669
Physical activity (min/week)	277 ± 337	287 ± 347	250 ± 311
Relationship status, *n* (%)			
Married	6268 (72.8)	4722 (73.2)	1546 (71.7)
*De facto*	414 (4.4)	286 (4.1)	128 (5.1)
Separated	217 (2.4)	132 (1.9)	85 (3.7)
Divorced	515 (5.1)	357 (4.7)	158 (6.1)
Widowed	528 (5.2)	468 (6.4)	60 (2.2)
Single	698 (10.0)	491 (9.6)	207 (11.1)
SEIFA	1025 ± 87	1026 ± 87	1023 ± 86
Level of education, *n* (%)			
Never to some high school	3445 (35.7)	2688 (37.7)	757 (30.7)
Completed university/equivalent	5195 (64.3)	3768 (62.3)	1427 (69.3)
Smoking status, *n* (%)			
Current	1338 (16.3)	921 (15.1)	417 (19.3)
Ex-smoker	2537 (25.9)	1938 (26.4)	599 (24.5)
Non-smoker	4765 (57.8)	3597 (58.5)	1168 (56.2)
History of CVD, *n* (%)	685 (6.7)	552 (7.5)	133 (4.5)
Prevalence of diabetes, *n* (%)			
Known diabetes	348 (3.3)	284 (3.9)	64 (2.0)
Undiagnosed diabetes	350 (3.5)	284 (3.9)	66 (2.4)
Impaired fasting glucose	512 (5.8)	387 (5.7)	125 (5.9)
Impaired glucose tolerance	1043 (10.3)	818 (10.7)	225 (9.3)
Normal glucose levels	6387 (77.1)	4683 (75.8)	1704 (80.4)
Perceived stress index	0.28 ± 0.16	0.20 ± 0.10	0.49 ± 0.10

Estimated using the survey command to apply the necessary weighting for selection bias. Values are mean ± SD unless otherwise stated. Lower stress represents Q1 to Q3; High stress represents Q4

*BMI* Body Mass Index, *CVD* Cardiovascular Disease, *SEIFA* Socio-Economical Index For Areas

### Associations of FV type intakes with high perceived stress

Compared to participants with the lowest intakes, those with the highest intakes of apples and pears, orange and citrus fruits, and bananas had a statistically significant 31, 25, and 24% lower odds, respectively, of having high perceived stress (Table 2). Similarly, each SD increase in consumption of apples (78 g/d) and bananas (35 g/d) was associated with 11 and 8% lower odds for perceived stress, respectively (Table 2). Mean perceived stress index was significantly lower in the higher intake quartiles of apples and pears, oranges and other citrus, and bananas (Online Resource 2).

**Table 2:** Odds ratios (OR) for high perceived stress by quartiles of specific types of fruit

	Per SD	Fruit intake quartiles			
		Q1	Q2	Q3	Q4
Apples and pears	76 g increase	*n* = 2162	*n* = 2165	*n* = 2153	*n* = 2160
Average intake (g/day)		7 (7, 8)	28 (27, 28)	66 (65, 66)	168 (162, 174)
High stress, *n* (%)		599 (34%)	582 (29%)	510 (25%)	453 (25%)
Model 1^1^	**0.86 (0.79, 0.94)**	Ref	0.81 (0.59, 1.13)	**0.64 (0.48, 0.86)**	**0.64 (0.51, 0.81)**
Model 2^2^	**0.89 (0.81, 0.97)**	Ref	0.76 (0.52, 1.10)	**0.68 (0.49, 0.94)**	**0.69 (0.53, 0.90)**
Orange and other citrus	50 g increase	*n* = 2162	*n* = 2165	*n* = 2153	*n* = 2160
Average intake (g/day)		2 (2, 2)	12 (12, 12)	34 (33, 34)	112 (108, 116)
High stress, *n* (%)		600 (31%)	610 (30%)	549 (29%)	425 (23%)
Model 1^1^	**0.90 (0.83, 0.97)**	Ref	0.98 (0.81, 1.19)	0.93 (0.74, 1.18)	**0.68 (0.56, 0.82)**
Model 2^2^	0.95 (0.89, 1.02)	Ref	0.88 (0.71, 1.01)	0.90 (0.71, 1.14)	**0.75 (0.64, 0.89)**
Bananas	35 g increase	*n* = 2166	*n* = 2161	*n* = 2155	*n* = 2158
Average intake (g/day)		4 (4, 4)	16 (16, 16)	36 (36, 37)	84 (82, 86)
High stress, *n* (%)		620 (33%)	604 (31%)	530 (27%)	430 (22%)
Model 1^1^	**0.83 (0.76, 0.90)**	Ref	0.89 (0.71, 1.13)	**0.74 (0.64, 0.85)**	**0.59 (0.51, 0.68)**
Model 2^2^	**0.92 (0.85, 0.98)**	Ref	0.93 (0.74, 1.19)	0.86 (0.72, 1.03)	**0**.**76 (0.64, 0.89)**

Odds ratios (ORs) and 95% confidence intervals (95% CI) obtained using the survey command for logistic regression. Fruit intake (g/d) is shown as mean and 95% CI. Lower stress represents Q1 to Q3; High stress represents Q4. Analyses were adjusted according to the following models: model 1^1^, unadjusted; and model 2^2^, multivariable-adjusted (confounding factors included age, sex, BMI [body mass index], energy intake, relationship status, physical activity, level of education, SEIFA [Socio-economical index for areas], smoking status, diabetes and history of cardiovascular disease). Numbers in bold are significantly different from Q1 (*p* < 0.05)

The associations between types of vegetables and high perceived stress are shown in Table 3. Compared to participants with the lowest intakes, those with the highest intakes of cruciferous, yellow/orange/red vegetables, and higher consumption of legumes (Q3) had a statistically significant 25, 27 and 26% lower odds of high perceived stress, respectively. Likewise, each SD increase in intake of cruciferous vegetables (10 g/d) and yellow/orange/red vegetables (25 g/d) was associated with 12 and 11% lower odds for perceived stress, respectively (Table 3). The mean perceived stress index was significantly lower in those with higher intake of cruciferous, yellow/orange/red vegetables and legume vegetables (Online Resource 3).

**Table 3:** Odds ratios (OR) for high perceived stress by quartiles of specific types of vegetables

	Per SD	Vegetable intake quartiles			
		Q1	Q2	Q3	Q4
Cruciferous vegetables	10 g increase	*n* = 2166	*n* = 2161	*n* = 2158	*n* = 2155
Average intake (g/day)		5 (5, 5)	15 (15, 15)	26 (26, 27)	51 (50, 52)
High stress, *n* (%)		610 (31%)	592 (31%)	520 (27%)	462 (23%)
Model 1^1^	**0.85 (0.77, 0.93)**	Ref	1.01 (0.84, 1.22)	0.84 (0.69, 1.02)	**0.67 (0.52, 0.87)**
Model 2^2^	**0.88 (0.80, 0.97)**	Ref	1.10 (0.90, 1.36)	0.91 (0.74, 1.13)	**0.75 (0.57, 0.99)**
Allium vegetables	6 g increase	*n* = 2210	*n* = 2114	*n* = 2167	*n* = 2149
Average intake (g/day)		1 (1, 1)	4 (4, 4)	7 (7, 7)	15 (14, 15)
High stress, *n* (%)		577 (29%)	511 (28%)	544 (26%)	552 (30%)
Model 1^1^	1.00 (0.89, 1.13)	Ref	0.95 (0.75, 1.20)	0.86 (0.68, 1.10)	1.05 (0.76, 1.47)
Model 2^2^	0.95 (0.84, 1.07)	Ref	0.90 (0.71, 1.13)	0.80 (0.62, 1.02)	0.90 (0.64, 1.27)
Yellow/orange/red vegetables	25 g increase	*n* = 2161	*n* = 2160	*n* = 2159	*n* = 2160
Average intake (g/day)		15 (14, 16)	32 (31, 32)	46 (45, 46)	76 (74, 77)
High stress, *n* (%)		579 (31%)	558 (29%)	535 (28%)	512 (25%)
Model 1^1^	**0.90 (0.83, 0.98)**	Ref	0.92 (0.75, 1.13)	0.86 (0.65, 1.14)	**0.76 (0.63, 0.93)**
Model 2^2^	**0.89 (0.83, 0.96)**	Ref	0.95 (0.77, 1.17)	0.86 (0.67, 1.11)	**0.73 (0.61, 0.88)**
Leafy green vegetables	10 g increase	*n* = 2168	*n* = 2165	*n* = 2151	*n* = 2156
Average intake (g/day)		4 (3, 4)	9 (9, 9)	15 (15, 15)	27 (27, 28)
High stress, *n* (%)		606 (31%)	569 (28%)	542 (28%)	467 (25%)
Model 1^1^	0.92 (0.85, 1.00)	Ref	0.85 (0.69, 1.04)	0.87 (0.75, 1.01)	**0.73 (0.57, 0.92)**
Model 2^2^	0.98 (0.90, 1.07)	Ref	0.86 (0.70, 1.05)	0.93 (0.78, 1.11)	0.85 (0.67, 1.08)
Legume vegetables	21 g increase	*n* = 2176	*n* = 2154	*n* = 2155	*n* = 2155
Average intake (g/day)		9 (9, 9)	21 (20, 21)	33 (32, 33)	59 (58, 60)
High stress, *n* (%)		599 (31%)	554 (29%)	488 (25%)	543 (29%)
Model 1^1^	0.95 (0.88, 1.04)	Ref	0.90 (0.70, 1.16)	**0.73 (0.56, 0.96)**	0.92 (0.75, 1.13)
Model 2^2^	0.93 (0.84, 1.02)	Ref	0.94 (0.76, 1.16)	**0.74 (0.59, 0.92)**	0.86 (0.70, 1.05)

Odds ratios (ORs) obtained using the survey command for logistic regression. Vegetable intake (g/d) is shown as mean and 95% confidence intervals. Lower stress represents Q1 to Q3; High stress represents Q4. Analyses were adjusted according to the following models: model 1^1^, unadjusted; and model 2^2^, multivariable-adjusted (confounding factors included age, sex, BMI [body mass index], energy intake, relationship status, physical activity, level of education, SEIFA [Socio-economical index for areas], smoking status, diabetes and history of cardiovascular disease). Numbers in bold are significantly different from Q1 (*p* < 0.05)

### Sensitivity and interaction analyses

To explore whether a healthy diet could be a confounding factor, we further adjusted the analyses for the DGI. Similar relationships were observed for types of FV intake and lower odds of high perceived stress (Q4 for apples and pears: OR [95% CI]: 0.72 [0.54, 0.95], orange and other citrus fruits: 0.77 [0.65, 0.92], bananas: 0.78 [0.68, 0.91], cruciferous: 0.77 [0.59, 1.01], yellow/orange/red vegetables: 0.78 [0.62, 0.92], and Q3 for legume vegetables: 0.75 [0.60, 0.94], compared to Q1).

We also further adjusted each fruit groups for other fruit groups (for example, the association of apples and pear with perceived stress was further adjusted for other non-apple and pears fruit). The relationship of all fruit types with lower odds of high perceived stress remained significant (Q4 for apples and pears: OR [95% CI]: 0.68 [0.48, 0.96], orange and other citrus fruit: 0.78 [0.64, 0.95], and bananas: 0.78 [0.65, 0.92]). Similarly, for vegetable types, we added other vegetables to the multivariable-adjusted model. Whereas the relationship of legumes and perceived stress remained significant (Q3 for legume vegetables: 0.80 [0.65, 0.98]), compared to Q1, the association of cruciferous and yellow/orange/red vegetables became non-significant (Q4: 0.85 [0.62, 1.16] and 0.89 [0.75, 1.05]), respectively, compared to Q1.

In separate interaction testing, sex and age did not influence the relationship between types of FV and perceived stress (sex: all *p*_interaction_ > 0.1, except *p*_interaction_ > 0.06 for orange and other citrus fruit and allium vegetables; age: all *p*_interaction_ > 0.1).

### Further analyses

There was a high correlation between vegetable diversity and total vegetable intake (rho = 0.8, *p* = 0.001), and when both variables were included in multivariable-adjusted models, high variable inflation factors (2.8–3.0) were recorded suggesting evidence for collinearity indicating that any association of vegetable diversity with lower odds of high perceived stress was largely related to the association resulting from total vegetable intake.

## Discussion

We have previously reported that higher FV intake is associated with lower perceived stress in adults aged ≥ 25 years participating in the AusDiab study [20]. We have now extended these findings and demonstrate that consuming specific types of fruit and vegetables are associated with lower odds of having high perceived stress. The groups with the highest consumption of apples and pears, oranges and other citrus, and bananas had 31, 25, and 24% lower risk of having high perceived stress, respectively, compared to those with the lowest intakes. For vegetables, cruciferous, yellow/orange/red and legumes were the main types driving the associations, with greater consumption being associated with 25, 27, and 26% lower odds of having high perceived stress. These relationships were robust and independent of a range of confounding lifestyle factors including a healthy diet and physical activity.

Prolonged stress is considered a risk factor for mental health problems such as anxiety and depression [1, 52], and modifiable risk factors, such as a healthy diet appear to be beneficial for mental wellbeing [12]. Although increasing evidence supports the existence of an inverse link between consumption of FV and mental health problems [12], the mechanisms to explain this relationship remain unclear. The protective role of fruit and vegetables on stress levels could be due to a range of their constituents, including minerals, vitamins, and other phytochemicals. Various phytochemicals found in specific FV have anti-oxidative and anti-inflammatory properties (i.e. carotenoids, vitamin K1 and flavonoids) that could potentially alleviate stress levels [19], contributing to the overall health benefits [53], including perceived stress. Apples and pears, and oranges and other citrus fruit, for example, are rich sources of flavonoids, dietary fibre, vitamins, carotenoids, and other minerals that seem to reduce inflammation and oxidative stress [54]. Likewise, the health benefits of bananas may be linked to their high content of tryptophan [55]; an essential amino acid (3) and precursor of serotonin, a neurotransmitter in the brain modulating mood [56].

For vegetable intake, similar associations were observed, where higher consumption of cruciferous, yellow/orange/red and legumes were associated with less perceived stress. These types of vegetables are rich in fibre, polyphenols and carotenoids which are linked to improvements in oxidative stress and inflammation [57]. Considering those with mental health problems have been shown to have higher oxidative stress and systemic inflammation [58], having a diverse intake of FV is likely to provide greater benefits due to a broader range of nutrients [59].

Of interest, the relationship of both cruciferous and yellow/orange/red vegetables with lower odds of high perceived stress was no longer evident after controlling for other vegetables (i.e. for cruciferous vegetables when other non-cruciferous vegetables were added to the model). These results add weight to the proposition that consuming a diverse range of vegetables is likely to have a positive influence on perceived stress. In contrast, for fruit, the associations of all fruit groups with lower odds of high perceived stress were independent of other fruit. This suggest that specific fruit such as apples and pears, oranges and other citrus, and bananas might be most beneficial. Collectively, such findings are similar to previous research considering other mental health outcomes [13].

A diverse consumption of FV has also been associated with better mental health in previous studies [13, 14]. Young women consuming lower amounts of citrus (OR [95%CI]: 3.14 [1.34–7.38] and green leafy (OR [95%CI]: 3.84 [2.05–7.19]) vegetables were more likely to present depressive symptoms [13]. In young adults, consumption of raw FV predicted lower depressive symptoms and better positive mood, life satisfaction, and flourishing [14]. This study identified the top 10 FV linked with improved mental health were bananas, apples, citrus, berries, grapefruit, kiwifruit, carrots, lettuce, cucumber, and green leafy vegetables (mainly spinach) [14]. Hence, the current daily recommendation for FV intake, whilst including a range of different types of FV (all colours of the rainbow) is likely to be most beneficial to alleviate stress, and consequently stress-related mental health problems. Future studies investigating the potential association between FV constituents (i.e., flavonoids) and perceived stress are needed to clarify the likely mechanism(s) for future interventions.

### Limitations and strengths of the study

Due to the cross-sectional nature of the study, we were unable to demonstrate direct causality or to make firm conclusions about the direction of the relationship. Since we also did not measure biomarkers of stress, such as cortisol, we were unable to confirm a relationship of FV types with such biomarkers. In addition, since vegetable diversity and total vegetable intake were very highly correlated, we were unable to determine if the beneficial associations of higher vegetable intake and greater vegetable variety with lower perceived stress were independent of one another.

Strengths of this study include the availability of information on intake of specific types of fruit and vegetables, the use of validated questionnaires collect data on dietary intake and perceived stress, and the adjustment for numerous demographic and lifestyle covariates to limit the influence of potential confounders. Future longitudinal studies are required to explore the causal directions of the observed associations, and to clarify their potential mechanisms.

## Conclusion

In Australian adults, higher consumption of specific types of FV was associated with lower odds for high perceived stress. These findings were independent of important potential confounding factors such as diet quality and physical activity. Specifically, we provide evidence for the benefits of specific types of FV (i.e. apples and pears, oranges and citrus, bananas, and cruciferous, yellow/orange/red and legume vegetables) when considering perceived stress. Based on these results, public health messages should continue to promote higher FV intake whilst also highlighting the importance of including ‘a rainbow of colours’ as part of a healthy diet.

## Supplementary Information

Below is the link to the electronic supplementary material.

Supplementary file1 (DOCX 59 KB)

Supplementary file2 (DOCX 19 KB)

Supplementary file3 (DOCX 21 KB)

## Data Availability

The data that support the findings of this study are available from the Baker Heart and Diabetes Institute. Restrictions apply to the availability of these data, which were used under license for the current study. Raw data are not publicly available. However, data described in the manuscript, codebook, and analytic code will be made available upon request and approval of the AusDiab Steering Committee.
