# Supplementary material for: Associations of specific types of fruit and vegetables with perceived stress in adults: the AusDiab study
Source: Eur J Nutr. 2022 Mar 20;61(6):2929–38. doi: 10.1007/s00394-022-02848-5 (PMC9363314; doi:10.1007/s00394-022-02848-5)
Supplement: Supplementary file 1 — Supplementary file1 (DOCX 59 KB) [file 394_2022_2848_MOESM1_ESM.docx]

**ELECTRONIC SUPPLEMENTARY MATERIAL – Online Resource 1**

**Associations of** **specific types of fruit and vegetables with perceived stress in adults: The AusDiab study**

Simone Radavelli-Bagatini*^1^, Marc Sim^1,2^, Lauren C. Blekkenhorst^1,2^, Nicola P. Bondonno^1^, Catherine P. Bondonno^1,2^, Richard Woodman^3^, Joanne M. Dickson^1,4^, Dianna J. Magliano^5,7^, Jonathan E. Shaw^6,7^, Robin M. Daly^8^, Jonathan M. Hodgson^1,2^, Joshua R. Lewis^1,2,9^

^1^Institute for Nutrition Research, School of Medical and Health Sciences, Edith Cowan University, Perth, WA, Australia

^2^Medical School, The University of Western Australia, Perth, WA, Australia

^3^Flinders Centre for Epidemiology and Biostatistics, Flinders University, Adelaide, SA, Australia

^4^School of Arts and Humanities (Psychology), Edith Cowan University, Perth, WA, Australia

^5^Diabetes and Population Health, Baker Heart and Diabetes Institute, Melbourne, VIC, Australia

^6^Clinical Diabetes and Epidemiology, Baker Heart and Diabetes Institute, Melbourne, VIC, Australia

^7^School of Public Health and Preventive Medicine, Monash University, Melbourne, VIC, Australia

^8^Institute for Physical Activity and Nutrition, School of Exercise and Nutrition Science, Deakin University, Geelong, VIC, Australia

^9^Centre for Kidney Research, Children's Hospital at Westmead, School of Public Health, Sydney Medical School, The University of Sydney, Sydney, NSW, Australia***Corresponding author:**

Simone Radavelli-Bagatini

Institute for Nutrition Research, School of Medical and Health Sciences, Edith Cowan University

270 Joondalup Drive, Perth, WA, 6027

Royal Perth Hospital (RPH) Research Foundation

Tel: +61 8 9224 0344

E-mail: s.radavellibagatini@ecu.edu.au

**
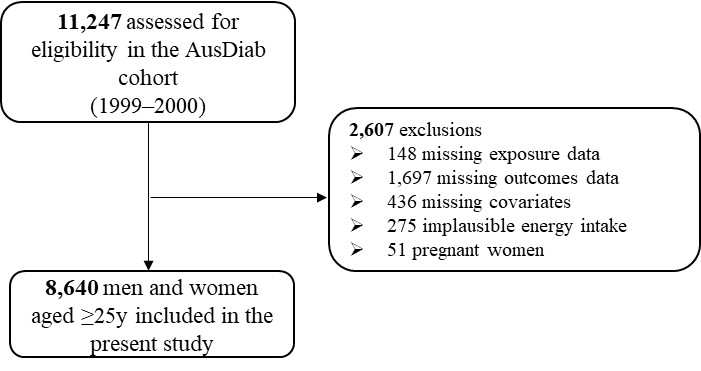
**

**Online resource 1.** Participant’s flow chart
