# Supplementary material for: Associations of specific types of fruit and vegetables with perceived stress in adults: the AusDiab study
Source: Eur J Nutr. 2022 Mar 20;61(6):2929–38. doi: 10.1007/s00394-022-02848-5 (PMC9363314; doi:10.1007/s00394-022-02848-5)
Supplement: Supplementary file 3 — Supplementary file3 (DOCX 21 KB) [file 394_2022_2848_MOESM3_ESM.docx]

**ELECTRONIC SUPPLEMENTARY MATERIAL – Online Resource 3**

**Associations of** **specific types of fruit and vegetables with perceived stress in adults: The AusDiab study**

Simone Radavelli-Bagatini*^1^, Marc Sim^1,2^, Lauren C. Blekkenhorst^1,2^, Nicola P. Bondonno^1^, Catherine P. Bondonno^1,2^, Richard Woodman^3^, Joanne M. Dickson^1,4^, Dianna J. Magliano^5,7^, Jonathan E. Shaw^6,7^, Robin M. Daly^8^, Jonathan M. Hodgson^1,2^, Joshua R. Lewis^1,2,9^

^1^Institute for Nutrition Research, School of Medical and Health Sciences, Edith Cowan University, Perth, WA, Australia

^2^Medical School, The University of Western Australia, Perth, WA, Australia

^3^Flinders Centre for Epidemiology and Biostatistics, Flinders University, Adelaide, SA, Australia

^4^School of Arts and Humanities (Psychology), Edith Cowan University, Perth, WA, Australia

^5^Diabetes and Population Health, Baker Heart and Diabetes Institute, Melbourne, VIC, Australia

^6^Clinical Diabetes and Epidemiology, Baker Heart and Diabetes Institute, Melbourne, VIC, Australia

^7^School of Public Health and Preventive Medicine, Monash University, Melbourne, VIC, Australia

^8^Institute for Physical Activity and Nutrition, School of Exercise and Nutrition Science, Deakin University, Geelong, VIC, Australia

^9^Centre for Kidney Research, Children's Hospital at Westmead, School of Public Health, Sydney Medical School, The University of Sydney, Sydney, NSW, Australia***Corresponding author:**

Simone Radavelli-Bagatini

Institute for Nutrition Research, School of Medical and Health Sciences, Edith Cowan University

270 Joondalup Drive, Perth, WA, 6027

Royal Perth Hospital (RPH) Research Foundation

Tel: +61 8 9224 0344

E-mail: s.radavellibagatini@ecu.edu.au

**Online Resource 3.** Association of perceived stress by quartiles of specific types of vegetables

|  | **Vegetable intake quartiles** | | | |
| --- | --- | --- | --- | --- |
|  | **Q1** | **Q2** | **Q3** | **Q4** |
| **Cruciferous vegetables** | *n=2,166* | *n=2,161* | *n=2,158* | *n=2,155* |
| *Average intake (g/day)* | 5 (5, 5) | 15 (15, 15) | 26 (26, 27) | 51 (50, 52) |
| ***Perceived stress index*** |  |  |  |  |
| *Model 1^1^* | 0.29 (0.28, 0.30) | 0.29 (0.28, 0.30) | **0.28 (0.27, 0.29)** | **0.26 (0.25, 0.28)** |
| *Model 2^2^* | 0.29 (0.27, 0.30) | 0.29 (0.28, 0.30) | 0.28 (0.27, 0.29) | **0.27 (0.26, 0.28)** |
|  |  | | | |
| **Allium vegetables** | *n=2,210* | *n=2,114* | *n=2,167* | *n=2,149* |
| *Average intake (g/day)* | 1 (1, 1) | 4 (4, 4) | 7 (7, 7) | 15 (14, 15) |
| ***Perceived stress index*** |  |  |  |  |
| *Model 1^1^* | 0.28 (0.27, 0.29) | 0.28 (0.27, 0.30) | 0.27 (0.26, 0.29) | 0.29 (0.27, 0.31) |
| *Model 2^2^* | 0.29 (0.28, 0.30) | 0.28 (0.27, 0.30) | 0.27 (0.26, 0.28) | 0.28 (0.27, 0.30) |
|  |  | | | |
| **Yellow/orange/red vegetables** | *n=2,161* | *n=2,160* | *n=2,159* | *n=2,160* |
| *Average intake (g/day)* | 15 (14, 16) | 32 (31, 32) | 46 (45, 46) | 76 (74, 77) |
| ***Perceived stress index*** |  |  |  |  |
| *Model 1^1^* | 0.29 (0.28, 0.30) | 0.28 (0.28, 0.29) | 0.28 (0.26, 0.30) | **0.27 (0.26, 0.28)** |
| *Model 2^2^* | 0.29 (0.28, 0.30) | 0.28 (0.28, 0.29) | 0.28 (0.27, 0.30) | **0.27 (0.26, 0.28)** |
|  |  | | | |
| **Leafy green vegetables** | *n=2,168* | *n=2,165* | *n=2,151* | *n=2,156* |
| *Average intake (g/day)* | 4 (3, 4) | 9 (9, 9) | 15 (15, 15) | 27 (27, 28) |
| ***Perceived stress index, mean*** |  |  |  |  |
| *Model 1^1^* | 0.29 (0.29, 0.30) | 0.28 (0.27, 0.29) | 0.28 (0.27, 0.30) | **0.27 (0.26, 0.28)** |
| *Model 2^2^* | 0.29 (0.28, 0.30) | 0.28 (0.27, 0.28) | 0.28 (0.27, 0.29) | **0.28 (0.26, 0.29)** |
|  |  | | | |
| **Legume vegetables** | *n=2,176* | *n=2,154* | *n=2,155* | *n=2,155* |
| *Average intake (g/day)* | 9 (9, 9) | 21 (20, 21) | 33 (32, 33) | 59 (58, 60) |
| ***Perceived stress index*** |  |  |  |  |
| *Model 1^1^* | 0.30 (0.29, 0.31) | **0.28 (0.27, 0.29)** | **0.27 (0.26, 0.28)** | **0.28 (0.26, 0.29)** |
| *Model 2^2^* | 0.29 (0.29, 0.30) | **0.28 (0.28, 0.29)** | **0.27 (0.26, 0.28)** | **0.28 (0.27, 0.29)** |

Values are shown as mean (95% CI). Estimated from the adjusted marginal means using survey command for linear regression with the types of vegetable quartiles as the exposure variable of interest. Analyses were adjusted according to the following models: model 1^1^, unadjusted; and model 2^2^, multivariable adjusted (confounding factors included age, sex, BMI [body mass index], energy intake, relationship status, physical activity, level of education, SEIFA [Socio-economical index for areas], smoking status, diabetes and prevalence of cardiovascular disease); Perceived stress is given as an index, ranging from 0 to 1 (lowest to highest). Numbers in bold are significantly different from Q1 (p<0.05).
